# Genetic variation associated with increased insecticide resistance in the malaria mosquito, *Anopheles coluzzii*

**DOI:** 10.1186/s13071-018-2817-5

**Published:** 2018-04-04

**Authors:** Bradley J. Main, Amanda Everitt, Anthony J. Cornel, Fereydoun Hormozdiari, Gregory C. Lanzaro

**Affiliations:** 1 Department of Pathology, Microbiology, and Immunology, UC Davis, Davis, CA 95616 USA; 2 Department of Entomology and Nematology, University of California, Davis, CA 95616 USA; 3 Department of Biochemistry and Molecular Medicine, MIND Institute and UC-Davis Genome Center, University of California, Davis, CA 95616 USA

**Keywords:** *Anopheles*, Malaria vector, Insecticide resistance, Adaptive introgression, *kdr*, P450, Carboxylesterase

## Abstract

**Background:**

Malaria mortality rates in sub-Saharan Africa have declined significantly in recent years as a result of increased insecticide-treated bed net (ITN) usage. A major challenge to further progress is the emergence and spread of insecticide resistance alleles in the *Anopheles* mosquito vectors, like *An. coluzzii*. A non-synonymous mutation in the *para* voltage-gated sodium channel gene reduces pyrethroid-binding affinity, resulting in knockdown resistance (*kdr*). Metabolic mechanisms of insecticide resistance involving detoxification genes like cytochrome P450 genes, carboxylesterases, and glutathione S-transferases are also important. As some gene activity is tissue-specific and/or environmentally induced, gene regulatory variation may be overlooked when comparing expression from whole mosquito bodies under standard rearing conditions.

**Results:**

We detected complex insecticide resistance in a 2014 *An. coluzzii* colony from southern Mali using bottle bioassays. Additional bioassays involving recombinant genotypes from a cross with a relatively susceptible 1995 *An. coluzzii* colony from Mali confirmed the importance of *kdr* and associated increased permethrin resistance to the *CYP9K1* locus on the X chromosome. Significant differential expression of *CYP9K1* was not observed among these colonies in Malpighian tubules. However, the P450 gene *CYP6Z1* was overexpressed in resistant individuals following sublethal permethrin exposure and the carboxylesterase gene *COEAE5G* was constitutively overexpressed*.*

**Conclusions:**

The significant P450-related insecticide resistance observed in the 2014 *An. coluzzii* colony indicates that ITNs treated with the P450 inhibitor piperonyl butoxide (PBO) would be more effective in this region. The known insecticide resistance gene *CYP6Z1* was differentially expressed exclusively in the context of sublethal permethrin exposure, highlighting the importance of tissue-specificity and environmental conditions in gene expression studies. The increased activity of the carboxylesterase *COEAE5G* in the resistant *An. coluzzii* colony suggests resistance to other insecticides like organophosphates. Additional gene expression studies involving other tissues (e.g. fat body) would provide a more comprehensive view of genes underlying metabolic insecticide resistance in *An. coluzzii* from Mali. Identifying genetic markers linked to these regulatory alleles is an important next step that would substantially improve insecticide resistance surveillance and population genetic studies in this important vector species.

**Electronic supplementary material:**

The online version of this article (10.1186/s13071-018-2817-5) contains supplementary material, which is available to authorized users.

## Background

Malaria deaths have been cut in half since 2000, due in large part to insecticide-treated bed net (ITN) campaigns and use in sub-Saharan African countries like Mali [1]. A major threat to further reductions in malaria incidents is the emergence and spread of insecticide resistance (IR) in *Anopheles* (*An*.) mosquitoes like *An. gambiae* and *An. coluzzii*: the primary mosquito vectors of malaria in Mali [2–4]. *Anopheles coluzzii* has been increasing in relative abundance coincident with the adaptive introgression of the knockdown resistance (*kdr*) allele from *An. gambiae* in 2006 and is now the major malaria vector in southern Mali [4–6]. *Kdr* refers to non-synonymous mutations in the voltage-gated sodium channel gene (*para*), which reduces pyrethroid-binding affinity; an insecticide resistance mechanism called target site resistance. The *kdr* allele has been increasing in geographical distribution and relative frequency throughout Africa, apparently in response to increased ITN use [7–9]. Detoxification genes like cytochrome P450 genes, carboxylesterases, and glutathione S-transferases (GSTs) can also confer resistance to insecticides [10–14]. For example, P450 genes like *CYP9K1* [15, 16], *CYP6P3* [12], *CYP6M2* [13], *CYP6Z1* [17], *CYP325A3* [18, 19], and others [20, 21] have been associated with insecticide resistance in anophelines. Insecticide resistance ultimately is the combination of multiple possible mechanisms including target site and metabolic resistance, as well as reduced cuticle penetrance [22, 23]. P450 genes and *kdr* may also act synergistically [24].

Genomic studies commonly focus on single nucleotide polymorphisms because they can be readily identified with short reads. However, there is evidence that structural variants, including inversions, duplications, and deletions may be involved in adaptation and speciation within the *Anopheles gambiae* complex. For example, distinct inversion states and microsatellite profiles on chromosome 2 have been associated with reproductively isolated populations of *An. gambiae*, previously identified as the Bamako, Savanna, and Mopti (i.e. *An. coluzzii*) chromosomal forms [25]. In *Drosophila*, P450 gene duplication has been linked to DDT resistance [26]. Thus, in this study, we examine structural and regulatory variation in an *An. coluzzii* population in southern Mali that is associated with increased insecticide resistance and a previously described selective sweep [4]. Due to the importance of tissue-specificity in gene expression assays [27] and active expression of the candidate P450 gene *CYP9K1* in Malpighian tubules [28], we used RNA-seq to examine expression differences between alternative haplotypes at *CYP9K1* (*cyp-1* and *cyp-2*) in Malpighian tubules. We also compared expression profiles after sublethal exposure to permethrin, as some genes in *An. gambiae* may be responsive to permethrin [29].

## Methods

### Mosquitoes

We assessed insecticide resistance in the following mosquito strains from Mali: 1995 *An. coluzzii* (MOPTI; MRA-763), 2014 *An. coluzzii* (Selenkenyi), and *An. gambiae* (pimperena; MRA-861).

### Insecticide resistance bioassays

To test if metabolic insecticide resistance (P450-related) and overall insecticide resistance has increased in the modern *An. coluzzii* population in southern Mali since the start of a major insecticide-treated bed net campaign in 2006, we performed insecticide resistance bottle bioassays, following Centers for Disease Control and World Health Organization guidelines [30, 31] on the 2014 and 1995 *An. coluzzii* colonies (and *An. gambiae* pimperena for reference). We tested the effects of permethrin alone and permethrin immediately after exposure to the P450 inhibitor piperonyl butoxide (PBO). In brief, mosquitoes were placed in a 250 ml Wheaton bottle coated with 400 μg of PBO (in an acetone solution) for 1 h. Then, the mosquitoes were transferred to another 250 ml Wheaton bottle coated with 21.5 μg of permethrin and knockdown mosquitoes were recorded every 15 min for 1 h. After these incubation periods, all living mosquitoes were placed in a pint-sized paper cage with constant access to 10% sugar solution. Then, mortality was assessed after 24 h. To estimate the effects of permethrin alone, the PBO treatment bottle was replaced with a control bottle coated with pure acetone. Deleterious effects from the experimental procedure of transferring mosquitoes between Wheaton bottles was assessed by replacing both the PBO- and permethrin-treated bottles with bottles treated with pure acetone.

### Individual mosquito bottle bioassays with F2 recombinant genotypes

We generated F2 mosquitoes by crossing 1995 *An. coluzzii* (*cyp-2* genotype at *CYP9K1*) virgin females with 2014 *An. coluzzii* (*cyp-1* genotype at *CYP9K1*) males to make F1 hybrids and then the F1 generation was allowed to interbreed. As *CYP9K1* is on the X chromosome, all F1 males were hemizygous *cyp-2*. In the F2 generation, female genotypes were expected to be a 50:50 mix of *cyp-1/2 and cyp-2/2.* F2 male genotypes were expected to be a 50:50 mix of hemizygous *cyp-1* or *cyp-2*. The permethrin resistance bioassays were conducted using 250 ml Wheaton bottles that were prepared as previously described. Between 10 am and 3pm, we placed each individual mosquito in a permethrin-treated bottle for at least 1 h and recorded when the individual was not able to stay upright or maintain normal flight (knockdown) upon disturbing the bottle and rotating it 360 degrees in the horizontal position. Immediately following knockdown, each individual was placed in a 1.5 ml tube and snap frozen in an aluminum block in dry ice and stored at -80 °C until DNA extraction.

### Transcriptome analysis following sublethal permethrin exposure

To induce expression of genes that may be responsive to permethrin [29], 3-day-old virgin females were exposed to a sublethal dose of permethrin (¼ × standard dose 21.5 μg/l) in a Wheaton bottle for 15 min. Control mosquitoes were exposed to acetone-treated bottles. Then mosquitoes were given a 4 h recovery period in a pint cage with constant access to a 10% sugar solution. This recovery time was chosen based on previous gene expression results following permethrin exposure in *An. gambiae* [29]. Mosquitoes were cold-anesthetized by placing the cage in a -20 °C freezer for 3 min immediately prior to dissections.

### Malpighian tubule dissection and RNA isolation

All dissections were performed between 1–4 pm to limit gene expression differences due to circadian rhythm genes. To isolate the Malpighian tubules, the penultimate abdominal segment of a cold-anesthetized female was gently pulled with a micro-tweezer until the gut, Malpighian tubules, and ovaries were isolated from the carcass. Following removal of the ovaries and foregut, the Malpighian tubules (and some hindgut) were then immediately transferred into lysis buffer (Zymo Research, Irvine, USA) and homogenized with a micropestle (Additional file 1: Table S1). Total RNA (and DNA in parallel) was then extracted from individual females or pools of 8–12 Malpighian tubules per biological replicate using the ZR-Duet kit (Zymo Research) with an on-column DNAse treatment step.

### Transcriptome sequencing and QC

We generated strand-specific mRNA-seq libraries (KAPA Biosystems, Wilmington, USA) for 6 biological replicates of control and treatment conditions for both the 1995 *An. coluzzii* (*cyp-2*) and 2014 *An. coluzzii* (*cyp-1*) genotypes (Additional file: Table S2). These 24 libraries were sequenced on a single lane of HiSeq3000 SR50 at the UC Davis DNA Technologies Core. Poor quality bases and Illumina adapter sequences were trimmed from the raw reads using Trimmomatic version 0.30 [32], with the following parameters: LEADING:3, TRAILING:3, SLIDINGWINDOW:4:15, MINLEN:36. The trimmed reads were then assessed for general quality using FastQC version 0.10.1 (Babraham Bioinformatics, Cambridge, UK).

### Read mapping and differential gene expression analysis

Single-end reads were mapped with STAR version 2.5 (--quantMode GeneCounts; [33]) to the *An. coluzzii* reference genome (scaffolds AcolM1) with gene annotation information (AcolM1.3.gtf; Additional file 1: Table S2). A detailed workflow of our differential expression analysis is available as a Jupyter notebook (Additional file 1: Table S3). In brief, gene level counts were converted to counts per million (cpm) using edgeR [34] and we removed genes with very low expression levels by requiring at least 5 libraries with a cpm > 2 for each gene. After this filtering step, we were left with 8485 genes that we tested for differential expression. We further normalized individual samples using the trimmed mean of M-values (TMM) method. To estimate differential expression between genotypes and between treatments, we used *voom* [35] in the *limma* package [36] to perform a variance-stabilizing transformation on the raw counts and then fit a linear model using *lmFit* [37]. We applied a cell means model including genotype and treatment after accounting for potential batch effects at the dissection stage. To identify differentially expressed (DE) genes we used the eBayes function [35] and Benjamini-Hochberg (FDR)-corrected *P*-values (< 0.05) from the *decideTests* function.

We classified a gene as a putative detoxification gene if it was included in the detoxification chip [18], which includes over 200 genes belonging to at least three major enzyme families: glutathione S-transferases, cytochrome P450 monooxygenases and esterases.

### Analyzing structural variation associated with post-2006 *An. coluzzii*

Structural variants were detected in whole genome sequencing data (Additional file 1: Table S4) using LUMPY version 0.2.13 [38]. Discordant paired-end and split-end reads were extracted using samtools. Library size, mean, and standard deviation were estimated using paired_distro.py script from LUMPY. The inferred breakpoints produced by LUMPY were genotyped using SVtyper version 0.1.2. Calls identified as break ends were removed and analyzed separately. Remaining inferred breakpoints were used if they were larger than 100 bp in size, were not homozygous reference in all samples, and had more than ten supporting reads in the two highest coverage samples. For remaining calls, the ratio of *cyp-1* samples having either a heterozygous or homozygous alternate to the total number of *cyp-1* samples possible was calculated. This was repeated for *cyp-2* samples, and Fisher’s exact test was performed to determine which SVs differed in frequency between the two populations.

Inferred breakpoints which corresponded to potential repeat elements and genic regions were determined using bedtools intersect version 2.17.0. For repeat elements, a 50% reciprocal overlap between structural variant call and repeat elements was required. For genic regions, one breakpoint must have occurred within 500 bp of gene.

## Results

### Testing for increased permethrin resistance in *An. coluzzii* from southern Mali

**Fig. 1:**
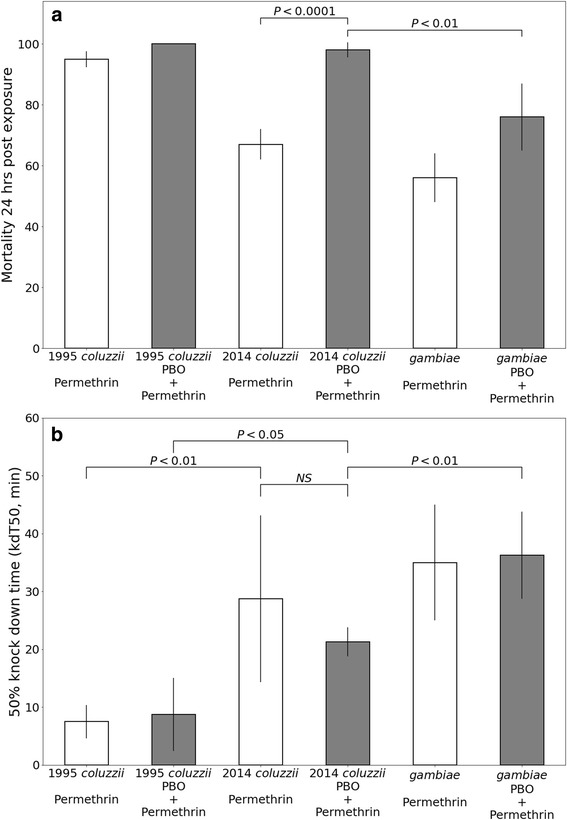
Estimating permethrin resistance related to P450 activity. **a** Mortality rates following permethrin exposure (white bars) and PBO + permethrin exposure (grey bars). The *Anopheles* genotype tested is indicated on the X-axis. Error bars indicate standard error. *P*-values were calculated using Fisher’s exact test. **b** Knockdown times following permethrin exposure (white bars) and PBO + permethrin exposure (grey bars). The Y-axis is the time (minutes) until 50% of the individuals were considered knocked down (kdT50). Each assay was performed with 4 replicate cages of 10 individuals each. Error bars indicate the 95% confidence interval. *P*-values were calculated using a t-test

According to the World Health Organization, a mosquito colony is considered resistant if less than 90% mortality is observed at 24 h post-exposure and susceptible if mortality is greater than 98% [31]. The 2014 *An. coluzzii* and *An. gambiae* colonies were both considered resistant, with 68% and 57% mortality after 24 h, respectively. The 1995 *An. coluzzii* colony was relatively susceptible with a mortality rate of 95% 24 h post-exposure to permethrin (Fig. 1a). To compare the knockdown phenotype in response to permethrin between colonies we estimated the knockdown times for 50% (kdT50) of mosquitoes in each bottle replicate. The mean kdT50 for 2014 *An. coluzzii* was 29 min, which was significantly longer (more resistant) than the 1995 *An. coluzzii* colony (7.5 min, t-test, *t*_(6)_ = 2.9, *P* = 0.02). Estimated knockdown times with permethrin exposure were not significantly different between 2014 *An. coluzzii* (29 min) and *An. gambiae* (35 min, t-test, *t*_(6)_= 0.66, *P* = 0.54; Fig. 1b).

To estimate permethrin resistance due to P450 activity, we compared mortality rates and knockdown times with and without pre-treatment of CDC bottles with the general P450 inhibitor piperonyl butoxide (PBO). Mortality significantly increased in the PBO-treated group of 2014 *An. coluzzii* from 67% (*n* = 43) to 98% (*n* = 53, Fisher’s exact test, *P* < 0.0001; Fig. 1a). Pre-treatment with PBO reduced the kdT50 for 2014 *An. coluzzii* from 28.75 to 21.25 min, but this change was not significant (t-test, *t*_(6)_=1.03, *P* = 0.34). Similar trends were observed in *An. gambiae*, but no significant PBO-effect was observed for mortality or kdT50 in the 1995 *An. coluzzii* colony (Fig. 1).

### Increased resistance in *An. coluzzii* is associated with a selective sweep on the X chromosome

**Fig. 2:**
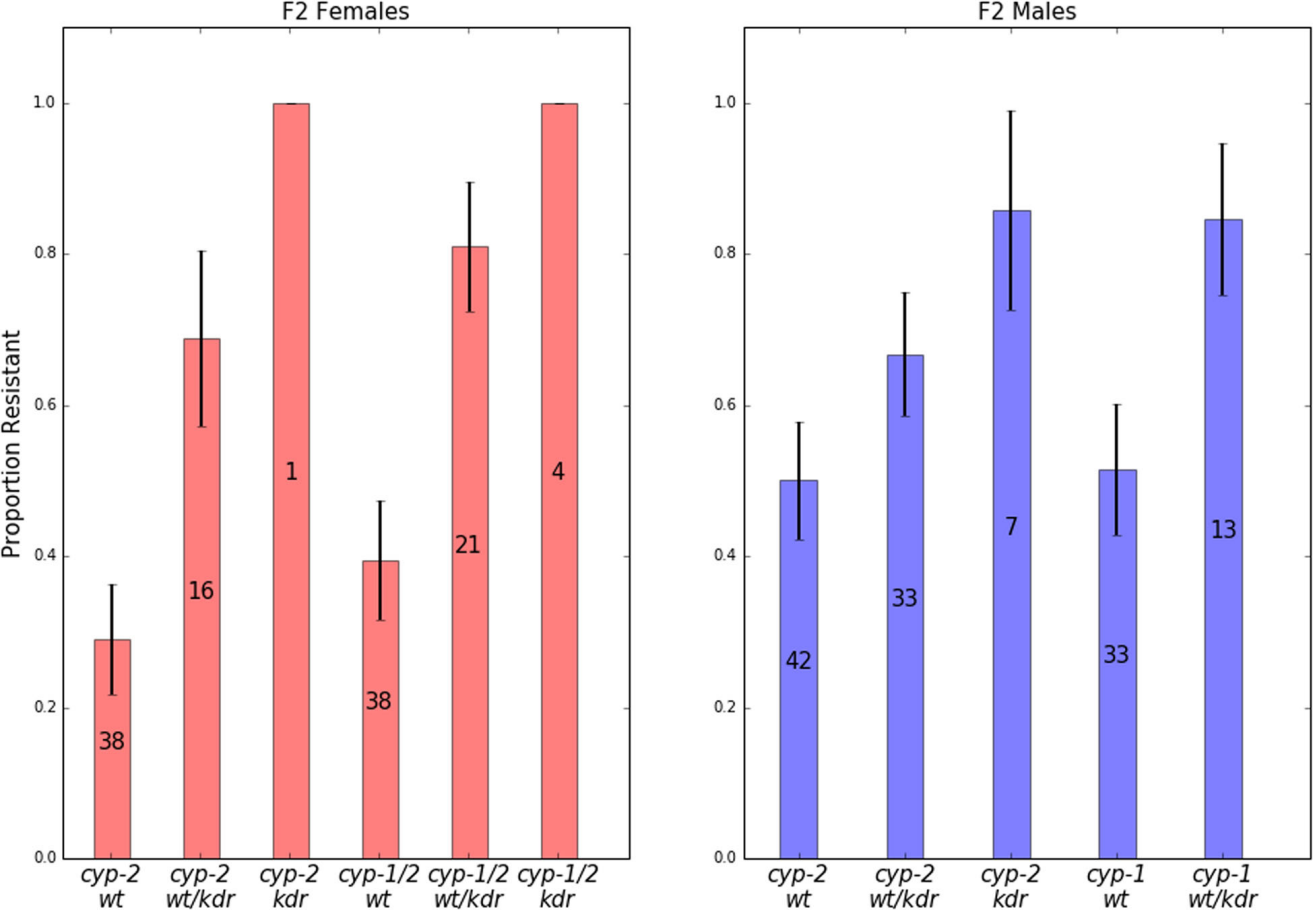
Permethrin resistance associated with a selective sweep on the X chromosome. Female (left) and male (right) F2 genotypes were individually tested in a CDC bottle bioassay for 60 minutes and then genotyped for *kdr* and the *cyp-1* or *cyp-2* haplotype at the *CYP9K1* locus. Individuals were grouped by genotype combination (X-axis) and the sample size is listed on each bar. The Y-axis is the proportion of individuals for each genotype/gender that were resistant to permethrin (mobile after 60 minutes of exposure)

A previous study identified multiple ~150 kb haplotypes at ~15 Mb on the X chromosome, namely *cyp-1*, *cyp-2*, and *cyp-3* [4]. After 2006, *cyp-1* became fixed in the population, resulting in a selective sweep. To test if genetic variation associated with the *cyp-1* haplotype confers insecticide resistance, we performed single-mosquito permethrin bottle bioassays on 122 female and 141 male F2 genotypes from a cross between *cyp-2* females (1995 *An. coluzzii*) and *cyp-1* males (2014 *An. coluzzii*). After the bioassay was completed, each mosquito was genotyped for *cyp* haplotype and *kdr*. Due to the direction of the initial cross, homozygous *cyp-1* genotypes were not generated. Individuals that survived beyond 60 min were considered resistant. There was a significant *kdr* effect on the proportion resistant in both males and females (*χ*^2^ = 24.99, *df* = 1, *P* < 0.0001, Fig. 2). In females, the *cyp-1* haplotype was associated with higher resistance (*χ*^2^ = 3.915, *df* = 1, *P* = 0.047, Fig. 2). This *cyp-1* effect was not observed in males.

### Genome structural variation in *An. coluzzii*

To investigate whether structural variation was selected for in *An*. *coluzzii* in addition to the *cyp-1* haplotype and *kdr*, we used the program LUMPY to call inferred breakpoints (edges of structural variants) genome-wide. We utilized 29 *An. coluzzii* genomes, including 17 *cyp-1* and 12 *cyp-2* individuals (Additional file 1: Table S4). After filtering, we identified 12,255 structural variants (SVs), including 9617 deletions, 1731 duplications, and 907 inversions (Additional file 1: Table S5). Of the SVs, 5739 overlapped with a repeat element and 5072 occurred within 500 bp of a known gene. A total of 66 SVs involve the *cyp-1* selective sweep region on the X chromosome. Most of these SVs (50/66) completely overlap the *cyp-1* haplotype region (~150 kb), while 6 SVs occur completely within the *cyp-1* haplotype region. A total of 223 SVs differed between the pre-2006 *cyp-2* and post-2006 *cyp-1* populations, using Fisher’s exact test. The 223 SV genes overlap 10,968 genes. However, if we exclude large (> 10 kb) SVs, 137 genes remained at or near (< 500 bp) SVs and the coding regions of 35 genes were overlapped by at least 1 bp (Additional file 1: Table S6). We did not identify known or putative insecticide resistance genes in these regions. To test for enrichment of these 137 genes in gene functional category, we performed a gene ontology analysis using g:profiler [39]. This is important because it is possible that the contribution of mutations involving several genes in the same biological pathway result in an overall adaptive phenotype (e.g. insecticide resistance). Enrichment was observed in the “regulation of Ras protein signal transduction” term (biological process) and three molecular functions: Rho guanyl-nucleotide exchange factor activity, protein binding, and molecular function regulator (Additional file 1: Table S7).

### Malpighian tubule-specific differential expression analysis

To elucidate genes underlying the increased insecticide resistance of the 2014 *An. coluzzii* colony, we compared gene expression profiles with the relatively susceptible 1995 *An. coluzzii* colony under control and sublethal permethrin exposure conditions. We generated 6 biological replicates of strand-specific mRNA-seq libraries from female Malpighian tubule tissue (see Methods) for each genotype and condition. The RNA-seq libraries were sequenced with single-end reads with a median of 15.5 million reads (Additional file 1: Table S2). Candidate genes that underlie increased insecticide resistance in the 2014 *An. coluzzii* colony were identified based on the assumption that resistance genes will be enriched among the total differentially expressed genes detected between colonies.

**Fig. 3:**
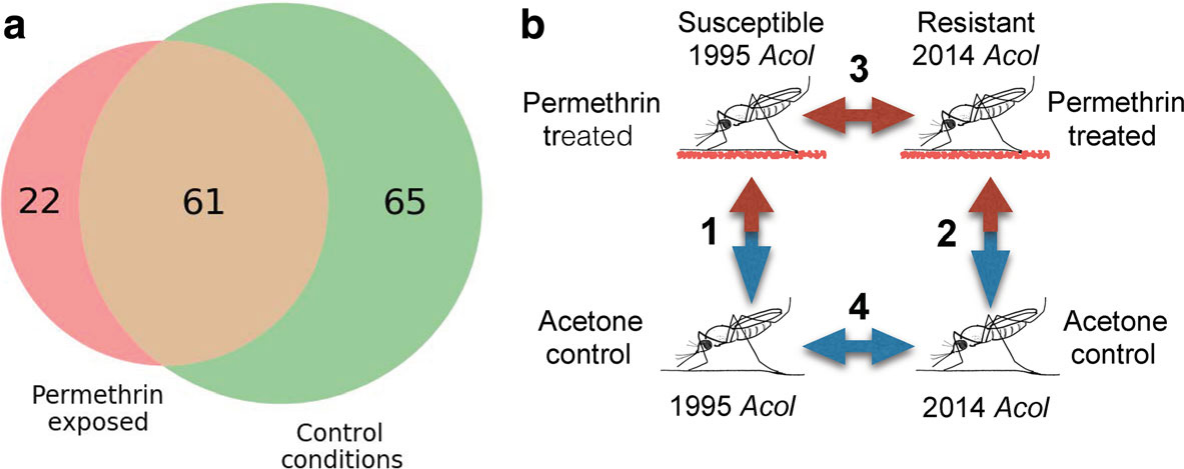
Differential expression analysis. **a** Numbers of differentially expressed genes in *An. coluzzii* Malpighian tubules associated with permethrin resistance following sublethal permethrin exposure and control conditions. Sixty-one genes are DE in both conditions. **b** We tested for differentially expressed genes between control and treatment conditions (1 and 2) and between *An. coluzzii* strains following sublethal permethrin exposure (3) and under control conditions (4)

A total of 8485 genes were tested for differential expression after requiring that a given gene was expressed above 2 counts per million in at least 5 biological replicates (Additional file 1: Table S8). Twenty-two differentially expressed genes between the colonies were unique to the treatment groups (Fig. 3a, b-3), 65 were unique to the control group comparison (Fig. 3a, b-4), and 61 were differentially expressed between the colonies under both treatment (Fig. 3b-3) and control conditions (Fig. 3a, b-4; Additional file 1: Table S9). We did not detect significant differentially expressed genes between control and sublethal permethrin treatment groups of either colony (Fig. 3b-1 and b-2).

The P450 gene *CYP9K1* was not differentially expressed between the colonies but tended toward under-expression in the 2014 *An. coluzzii* colony. Under control conditions the log fold change (FC) was -0.56 (*P* = 0.32) and under treatment conditions the logFC was -0.04 (*P* = 0.96), indicative of underexpression of the *cyp-1* allele (2014 *An. coluzzii*) compared to the *cyp-2* allele (1995 *An. coluzzii*). We also detected under-expression of the *cyp-1* allele in individual female F1 hybrid Malpighian tubules under control [Log2 (*cyp-1*/*cyp-2*) = -0.74] and permethrin-treated conditions [Log2 (*cyp-1/cyp-2*) = -0.84] (Additional file 1: Table S10).

We performed a gene functional enrichment analysis of all differentially expressed genes between the colonies, including control and treatment conditions using g:profiler [39]. This test resulted in an enrichment of genes in the steroid biosynthesis pathway (KEGG:00100) and GO terms related to several metabolic processes, glucuronidation, and flavonoid biosynthesis (Additional file 1: Table S11). There was no overlap between polymorphic structural variants (i.e. indels < 10 kb) and the differentially expressed genes detected in this study.

### Detoxification genes

Three of the DE genes between the 2014 *An. coluzzii cyp-1* colony and the 1995 *An. coluzzii cyp-2* colony under treatment conditions and three DE genes under control conditions were known detoxification genes. The carboxylesterase gene *COEAE5G* was upregulated in the resistant 2014 *An. coluzzii cyp-1* colony in both permethrin-treated (logFC 1.68, adj. *P* < 0.001) and control conditions (logFC 1.07, adj. *P* < 0.01). The P450 gene *CYP6Z1* was upregulated in the resistant 2014 *An. coluzzii cyp-1* colony (logFC 0.94, adj. *P* < 0.05) and the glutathione S-transferase (*GSTu2*) was downregulated in the 2014 *An. coluzzii cyp-1* individuals (logFC -1.39, adj. *P* < 0.05) in the context of sublethal permethrin exposure. Detoxification genes that were DE in the control conditions included the P450 gene *CYP4H19* (logFC -2.04, adj. *P* < 0.01) and the *thioredoxin peroxidase 4* (*TPX4,* logFC -2.05, adj. *P* < 0.05).

## Discussion

Using bottle bioassays we demonstrate that the 2014 *An. coluzzii* colony was more resistant than the 1995 *An. coluzzii* colony in terms of knockdown time and mortality at 24 hours. PBO assays indicated that P450 genes confer added insecticide resistance to the 2014 *An. coluzzii* colony. High susceptibility in the 1995 *An. coluzzii* colony inhibited our ability to estimate the contribution of P450 genes to resistance in this colony.

Previous studies have identified a selective sweep on the X chromosome of *An. coluzzii* associated with increased relative fitness [4]. CDC bottle bioassays on individual F2 recombinant individuals confirm the known role of *kdr* in permethrin resistance and support a link between the selected haplotype (*cyp-1*) and increased resistance.

This is the first study to examine structural variation within *An. coluzzii* populations. This is important because it is known that insects can gain insecticide resistance *via* copy number variation [26]. The SVs that were polymorphic between pre- and post-2006 *An. coluzzii* occurred at or near 137 genes. None of these genes are known detoxification genes [18]. At least one SV occurred in the intron of *kdr*, potentially affecting gene expression levels, but we did not detect differential expression of the *para* gene.

Among genes that may be affected by the polymorphic SVs, we detected an enrichment of genes in the “regulation of Ras protein signal transduction” biological process and the Rho guanyl-nucleotide exchange factor activity molecular function. Ras proteins are small GTPases that are involved in general signal transduction and cell growth. Rho guanyl-nucleotide exchange factor activity is involved in the exchange of guanyl nucleotides for a Rho GTPase. Further studies are needed to test if and how specific signal transduction genes (i.e. GTPases) are affected by SVs that may be under selection in these *An. coluzzii* populations.

Multiple studies have explored gene expression differences associated with insecticide resistance by grinding up whole body individuals from a colony that has been identified as resistant or susceptible. However, this approach may overlook genes that are highly tissue-specific [28] or that are responsive to environmental stimuli, like permethrin exposure [29]. As testing multiple tissues was beyond the scope of this study, we focused on expression differences in Malpighian tubules because this tissue is important for detoxification and the candidate P450 gene *CYP9K1* has been shown to be highly expressed in this tissue [28]. This approach increased the sensitivity to detect differences in genes that are active in Malpighian tubules (and attached hindgut), while decreasing our ability to identify DE genes that are more active in other tissues, like the fat body.

The carboxylesterase gene *COEAE5G* is constitutively overexpressed in the resistant *cyp-1* 2014 colony compared to the relatively susceptible c*yp-2* 1995 *An. coluzzii* colony and the logFC increased under sublethal permethrin exposure conditions (logFC 1.07 *vs* 1.68). Thus, the *COEAE5G* allele in the resistant *An. coluzzii* may also be more responsive to permethrin exposure. Further examination of *COEAE5G* activity in different resistant *An. coluzzii* colonies, tissue types and environmental conditions and its association with insecticide resistance is needed to assess the relative importance of *COEAE5G* in elevated insecticide resistance in Mali.

The P450 gene *CYP4H19* was downregulated in the *cyp-1* colony under control conditions. While resistance genes are commonly upregulated, P450 gene activity can have toxic effects [40] and downregulation of P450 genes has been previously reported in resistant colonies [18, 41]. Interestingly, the antioxidant *TPX4* was also downregulated in the *cyp-1* colony by more than two-fold under control conditions. Downregulation of *TPX4* was also observed in the DDT resistant colony ZAN/U but not the RSP resistant strain [18]. Thus, insecticide resistance involves multiple mechanisms, which may have distinct compensatory strategies, including downregulation of particular genes.

The P450 gene *CYP6Z1* has been shown to metabolize permethrin [17, 42–44] and was upregulated in the *cyp-1* resistant *An. coluzzii* colony following sublethal permethrin exposure, but not under control conditions. This indicates that genetic variation in the *cyp-1* colony increases the responsiveness of *CYP6Z1* to permethrin exposure. This is an important finding because candidate insecticide resistance genes like *CYP6Z1* can be missed with typical approaches involving whole body mosquitoes under standard rearing conditions. Additional tissue-specific gene expression studies in hybrid individuals would elucidate whether this regulatory variation is at *CYP6Z1* (*cis*) or associated with upstream transcription factors (*trans*).

## Conclusions

We detected complex insecticide resistance in *An. coluzzii* from southern Mali, supporting the use of PBO in bed nets in this region. The P450 gene *CYP6Z1* was overexpressed in Malpighian tubules of resistant individuals following sublethal permethrin exposure. This finding highlights the importance of tissue-specificity and environmental conditions in gene expression studies. The carboxylesterase gene *COEAE5G* was constitutively overexpressed in resistant *An. coluzzii*, suggestive of resistance to other insecticides like organophosphates. Bioassays involving recombinant genotypes confirmed the importance of *kdr* and the *cyp-1* haplotype at the *CYP9K1* locus in knockdown resistance to permethrin. However, significant differential expression of *CYP9K1* was not observed in Malpighian tubules. Additional expression studies involving other tissues (e.g. fat body) would provide a more comprehensive view of genes underlying metabolic insecticide resistance in *An. coluzzii* from Mali. Identifying genetic markers linked to these regulatory alleles is an important next step that would substantially improve insecticide resistance surveillance and population genetic studies in this important vector species.

## Additional file

Additional file 1: **Table S1.** This table provides links to video examples of the Malpighian tubule dissections described in the methods. **Table S2.** Illumina sequencing read depths per mRNAseq library and associated metadata. **Table S3.** A Jupyter notebook describing the differential expression workflow. **Table S4.** Genome coverage data. **Table S5.** Genome-wide structural variant calls in *An. coluzzii*. **Table S6.** A list of genes that were overlapped by a structural variant (< 10kb). **Table S7.** Gene ontology results from genes in table S5. **Table S8.** mRNAseq count data for each gene and corresponding *An. gambiae* ortholog. **Table S9.** All differentially expressed genes. **Table S10.** This Jupyter notebook describes the allele-specific expression analysis of *CYP9K1* in a *cyp-1*/*cyp-2* F1 hybrid. **Table S11.** Gene ontology results. (XLSX 3557 kb)

## Abbreviations

WHO: World Health Organization
ITN: insecticide-treated bed net
kdr: knockdown resistance
CDC: Center for Disease Control
kdT50: knockdown time for 50% of the sample
DE: differential expression
SV: structural variant
GO: Gene Ontology
PBO: piperonyl butoxide
CI: confidence interval
